# Sámi Healthcare Staff Experiences of Encounters with Sámi Patients and Their Expectations for Non-Sámi Healthcare Staff

**DOI:** 10.3390/nursrep14040201

**Published:** 2024-10-01

**Authors:** Tove Mentsen Ness, Grete Mehus

**Affiliations:** 1 Leader Centre for Sámi and Indigenous Studies, Faculty of Education and Art, Nord University, 7601 Levanger, Norway; 2 Faculty of Health Science, UiT The Arctic University of Norway, 9601 Hammerfest, Norway; grete.mehus@uit.no

**Keywords:** Sámi healthcare staff, experiences, expectations, Sámi patients, “two-eyed seeing”, nursing

## Abstract

Objective: The aim of this study was to explore Sámi healthcare staff experiences in encounters with Sámi patients and their expectations for non-Sámi healthcare staff. Methods: Focus groups and individual interviews with 14 participants were conducted, and the participants’ experiences were subjected to a thematic analysis approach. Results: The findings show that the Sámi healthcare staff expect non-Sámi healthcare staff to enhance their knowledge about Sámi language and culture. The Sámi healthcare staff also expect non-Sámi healthcare staff to gain knowledge of diversities within the Sámi cultures. Additionally, the results show that the participants felt that the encounters were more authentic when the patients had the same background as themselves. Conclusion: These results were also based on the participants’ experience of resistance from non-Sámi healthcare staff, which can be addressed by the non-Sámi healthcare staff and healthcare institutions enhancing their knowledge of Sámi history, culture, and language. This can be conducted by making efforts to meet the participants’ expectations and experiences, enhancing their knowledge of Sámi history, culture, and language, and showing respect to provide culturally safe care. Further, tacit knowledge and what can be seen as “two-eyed seeing”, as demonstrated by the participants, are not necessarily transferable to non-Sámi healthcare staff. Despite this, all healthcare staff, through experience, recognition, and reflections on encounters with Sámi patients and Sámi healthcare staff, can develop awareness in what is addressed as “two-eyed seeing” by the participants in this study.

## 1. Introduction 

Sápmi is the homeland of the Sámi population, extending across parts of Norway, Sweden, Finland, and Russia. The population of Sápmi is approximately 95,000, most of whom live in the Norwegian part of Sápmi [1]. Due to a strong colonisation process, especially from 1850 onwards, several Sámi have lost their mother tongue and connection to Sámi culture. The assimilation is almost complete, especially in sea Sámi areas of Norway [2,3]. 

In Norway, healthcare services are offered to all in need of care, irrespective of their cultural background or where they live [4,5,6,7]. This means that care recipients with a Sámi background have the right to receive healthcare services adapted to their cultural and linguistic background, according to the Sámi Act (12 June 1987), the ILO Convention No. 169 on Indigenous Rights, and the Patients’ Rights Act, § 3-2 and 3-5, although this is not yet the case [8,9,10].

Qualitative studies have revealed that Sámi patients, both young and older, feel neglected in terms of the use of the Sámi language in care, lack of interpreting services, and accessing medical information to enable them to understand and agree to treatment options, e.g., [11]. This is often due to the healthcare personnel’s lack of competence in Sámi language and culture [12]. Mehus et al. [13] stress that when personnel fail to understand the symptoms and experiences of a Sámi care recipient, the person could interpret this as disrespect or failure to understand their identity and background. Clearly, Sámi patients are at greater risk in hospitals far away from their local community [12,13,14,15]. 

Research from all over Sápmi indicates that the welfare system does not offer healthcare adapted to Sámi values and cosmology and that the same standardised healthcare services developed for the majority population are offered to all [13,14,16,17,18,19]. 

The perspectives of healthcare staff on this are that there is a lack of discussion and focus on Sámi culture, art, and music in clinical care [12,20,21,22], and this may place Sámi patients in a culturally unsafe environment in encounters with healthcare and thus make them feel culturally insecure [13]. Another study from the South Sámi area found that nurses working in home care in this area reported that they treated everyone in the same way, regardless of the care recipients’ ethnic identity and, therefore, their cultural and language needs [16]. This is not desirable in terms of culturally safe care, where healthcare staff have to be aware of their power positions based on (a) their privileges as representatives of the majority population and (b) professional positions and representatives of the Western healthcare system, which is also connected to the (c) governmental oppression of the Sami people back in the days of active assimilation policy [2].

One study also reveals that some healthcare staff do not see the need for interpreting when patients are bilingual because they think that “the patients speak Norwegian well enough” [12]. This may be because healthcare staff tend to relate to everybody “in the same way” and may therefore discriminate against patients in various ways, even if that was not their intention. Larsen et al. [23] demonstrate that it may be difficult for non-Sámi healthcare staff to reach the Sámi population with public health services. To achieve this, members of the minority and the majority groups must discuss the appropriate ethnic and ethno-political positions that non-Sámi and Sámi healthcare staff may represent, to facilitate healthcare services for the Sámi, the Indigenous population in Norway. 

Studies show that Sámi patients feel insecure with people who do not understand them, both linguistically and culturally [12,14]. However, some studies report a more nuanced picture; Dagsvold et al. [4] and Ness et al. [24] stress that not all Sámi patients find it essential to speak Sámi in healthcare encounters, stressing that the competence of the healthcare staff is more important than their Sámi background and ability to speak Sámi [25]. 

In sum, healthcare services in Norway should offer a more culturally safe care practice which must be ethical, helpful, and welcoming to Sámi patients regarding all their needs revealed in previous research. Adapting healthcare services better to the Sámi cultures in Norway is more likely with the new health education curricula in Norway from 2019 with mandatory learning outcomes addressing Sámi issues for all students, which will enhance the knowledge of Sámi cultures, languages, and ways of life for healthcare personnel [26,27,28]. 

The principle of cultural safety in clinical practice involves expectations for healthcare staff to include social justice, trust, respect, self-awareness, and self-reflection in their practice [29]. They must thus be aware of their power position and make sure that all patients feel safe and that there is no institutional racism or group or personal racism in healthcare encounters [14,30]. Cultural safety and person-centred care are two closely related terms. If staff caring for Sámi patients are person-centred, respectful, and self-reflective on their power position and privileges as healthcare representing the Western medicine system, this might be expected to lead to a culturally safer environment, regardless of whether the personnel have an Indigenous background or a high level of academic and medical knowledge. All patients need to be seen as the person they are, which includes cultural needs, their preferred language, and the kind of communication and care situation they desire; they should not have to feel resistance or neglect in this matter [29,31,32]. In this study, we therefore wanted to explore how Sámi healthcare staff experience encounters with Sámi patients and their minimum expectations for non-Sámi healthcare staff. The aim of this study was therefore to explore Sámi healthcare staff experiences in encounters with Sámi patients and their expectations for non-Sámi healthcare staff. 

## 2. Method

### 2.1. Design

This is a hermeneutic phenomenological study based on interviews with 14 participants [33], with the aim of exploring Sámi healthcare staff experiences in encounters with Sámi patients and their expectations for non-Sámi healthcare staff. Data were collected in focus group interviews [34] and a few individual interviews [35] in order to elicit the participants’ experiences. Patton [35] emphasises that it is important to try to understand the world from the participants’ viewpoint, which means that one has to see the uniqueness in all the participants’ perspectives and enable their contribution to shape the formation of new knowledge [33]. 

### 2.2. Participants and Sampling Procedure

During spring 2022, 14 healthcare staff aged between 32 and 68 years with a Sámi background were interviewed by the first author (TMN). Eleven women and three men participated in this study. Most of the participants had a Lule Sámi or South Sámi background, while a few had a North Sámi background, and all had mostly worked in areas where Sámi patients are considered a minority in healthcare settings. All participants had experience as healthcare staff, and their professions included registered nurse, state enrolled nurse, and doctor. The participants had work experience in municipal long-term care settings and specialised healthcare in local or central hospitals. Several had worked in various positions in healthcare. Some met Sámi patients occasionally in their work, while others had worked specifically with Sámi patients during most of their career as healthcare staff. The inclusion criteria were that they were Sámi and that they had experience of encountering Sámi patients. All participants self-identified as Sámi.

Recruitment was conducted in two ways. Firstly, the first author’s (TMN) acquaintances in the Sámi community provided names and phone numbers of potential participants. Secondly, the first author talked about this study at different conferences where possible participants could be present; some of these then joined this study but also provided the names of other possible participants who later agreed to participate. 

### 2.3. Focus Group Interviews and Individual Interviews

In this study, focus groups were conducted to facilitate an environment that enabled the participants to talk about certain selected topics. This encouraged participants to share perceptions and views without feeling pressure to reach a consensus [34]. Krueger and Casey [34] stress that when this is conducted several times with different participants, the researcher can identify trends and patterns in the perceptions of the participants. 

There were five focus group interviews with thirteen participants; the size of the focus groups varied from two to four participants. There were three follow-up interviews afterwards with some of the participants. Two of these were held because of time constraints for the participants, while one was because of an unexpected event for a participant. One individual interview was conducted with a participant unable to attend any of the focus group interviews. Three focus group interviews were face-to-face, while one was on Teams. The final focus group interview was a hybrid, where one participant used Teams, while the other two met the first author face to face. Because the participants mostly lived in sparsely populated areas far from each other, and several were working shifts, it was difficult to arrange face-to-face interviews. The focus group interviews lasted from 60 to 90 min, while the individual interviews took from 35 to 72 min. All interviews were conducted and transcribed verbatim by the first author. The participants were asked to talk about what they experienced as healthcare personnel in encounters with Sámi patients and what they saw as essential in these encounters. 

### 2.4. Data Analysis

In order to analyse the data, a thematic approach was chosen, inspired by Braun and Clarke [36], and Braun et al. [37]. Braun et al. [37] emphasise that thematic analysis is a possible method to adopt that offers flexibility when using both individual interviews and focus groups. In the first phase of the analysis process, the authors must familiarise themselves with the data, and this was conducted by both authors as they read all transcripts several times, focusing on the participants’ experiences and expectations. In this phase, both authors made preliminary notes about what attracted their attention and in order to understand the textual material. After this, in the second phase, the authors met several times digitally on Teams and used an inductive approach to the data when coding, letting the data speak, in other words, a bottom–up approach to identifying the meaning and what the data were saying. This entailed numerous discussions between the authors about what attracted their attention. The data were coded, first individually and then together, in addition to being discussed. This new phase of coding led to the construction of themes where the previous coding formed the basis. In this phase, the themes are built, moulded, and developed in relation to the research question at hand, according to Braun et al. [37]. The next phase is called revising and defining themes, and here it is important to compare all the coded data with each other in order to determine whether they overlap or fit the entire dataset [37]. In this phase, it is essential to gain a nuanced and in-depth understanding, and this was conducted through numerous discussions between the authors, leading to greater clarity and a consensus regarding themes. The research question, containing the expectations and experiences of the participants, was always kept in mind in the analysis process and in the process of reaching a consensus regarding themes. The authors went back and forth reflecting over previous notes and the entire dataset until a consensus was reached. 

### 2.5. Ethical Considerations and Roles in the Research Team

Informal oral consent for participation was deemed sufficient, and participants were informed about the possibility of withdrawing at any time during this study, an option that no one exercised. Informal oral consent was obtained from the participants and anonymity was guaranteed before they took part in this study. To protect their anonymity, no real names were transcribed from the interviews. Most of the data were collected from small rural communities, which can be transparent and identifiable, and to protect the participants, we therefore had to ensure that narratives, situations, cases, and descriptions would not be recognised. Therefore, we reconstructed some of the stories based on gender, places, and objects that could reveal the participants’ identity, which is a typical challenge in research in transparent rural communities [38,39]. Instead, general titles and identification numbers such as healthcare personnel one, two, etc., were used. This study was assessed by the Norwegian Agency for Shared Services in Education and Research (SIKT) (No. 788125) and conducted in accordance with the Declaration of Helsinki [40]. Since this study was planned and carried out prior to the creation of the expert ethical committee for Sami health research in 2019, this study did not ask for permission from the Sami Parliament. In addition, this study aimed to collect experiences from Sámi healthcare staff, not Sámi patients. Therefore, this study would not require approval from the Regional Committees for Medical and Health Research Ethics or the Sami Parliament. Standard research ethical procedures have been followed. The first author (TMN) collected and transcribed the data, but both authors contributed to the analysis of the data and the writing and revision of this article. Both authors (TMN and GM) are experienced, privileged, registered nurses and teachers and have conducted several qualitative studies in Sápmi, all based on interviews. 

The first author (TMN) is Norwegian, and the other has a mixed ethnic background (GM). The interviews were conducted in Norwegian because the participants speak various Sámi languages and because some of the participants do not speak Sámi and neither does the first author. The first author (TMN) has conducted all her previous research studies and interviews in Norwegian. 

## 3. Findings

The analysis revealed two themes with two associated sub-themes, see Table 1 below.

### 3.1. Various Expectations for Non-Sámi Healthcare Staff

#### 3.1.1. Knowledge About Sámi Cultures and Language

The participants talked about their own and their Sámi patients’ expectations for non-Sámi healthcare staff. Several said that the Sámi patients they had met felt that they had to educate non-Sámi healthcare personnel in care situations and spend the time on this when they should be receiving healthcare, resulting in less time to receive care and treatment for their health issues. Some participants also talked about a lack of knowledge among their non-Sámi colleagues, even when these colleagues lived in areas with a large Sámi population, e.g., with reindeer husbandry. This made the participants feel discouraged, as one explained:

“…one of my colleagues who lives in an area where there’s reindeer herding asked me, ‘Where are the reindeer during the winter? Are they in barns?’ That question tells you quite a bit. So why should she relate to a Sámi patient any differently? She doesn’t have any background knowledge”.

Even though many non-Sámi healthcare personnel lack knowledge about Sámi culture and language, some of the participants highlighted that all healthcare personnel have good intentions, and one stated the following: “Of all the healthcare professionals I’ve met, I’ve never met one who doesn’t want the best for their patients”.

Even with these good intentions, several participants underlined that non-Sámi healthcare personnel must acquire more general knowledge about Sámi culture and language to improve healthcare for Sámi patients. One participant exemplified this as follows:

“It’s not the patient’s responsibility to talk about their background. I think you [healthcare personnel] must know what your responsibility as healthcare personnel is”.

All participants in this study talked about knowledge in encounters with Sámi patients. Several explained how it could be to communicate with older Sámi patients and what their non-Sámi colleagues should be aware of, for example, the following:

“…they nod and smile and are always grateful for everything, many Sámi patients do that, and then things can easily go wrong. And when you ask the patient, is it okay if I do this? And he says yes, or he doesn’t answer, so then you think it’s ok, but you must understand that communication is so different. He has so much respect for authorities like a doctor or nurse, so you must know that…//…Another thing is that you must see the family as a resource. And sometimes they want you to talk to their family instead of them as patients, and this can be a good idea, because you don’t necessarily get honest feedback from older patients”.

This demonstrates that healthcare personnel cannot always assume that Sámi patients will express what they think and that family members therefore could sometimes bridge the gap in understanding and should thus be involved in decisions related to the patient. A few participants also implied that the Norwegianisation process has formed the way older Sámi patients communicate in healthcare encounters, such as being cautious and not always saying what they mean. 

Several participants emphasised the importance of having knowledge of Sámi language, especially for older patients, and one participant said the following:

“For the older generations the Sámi language has great significance, so if you learn some Sámi words as non-Sámi healthcare personnel, that has great importance for them, because then you can use some simple words and greet them in Sámi”.

Several also emphasised the importance of family and how the whole family might turn up to be with a Sámi patient admitted to a hospital or nursing home. One explained that they sometimes met healthcare staff who wondered why there were so many family members, and one had once asked if the patient was a very important person, and they replied, “She’s Sámi”, meaning that being a Sámi entails the whole family coming to be with the patient, which is part of the general knowledge non-Sámi healthcare personnel need to be aware of. 

All participants in this study emphasised the importance of non-Sámi healthcare personnel having general knowledge about Sámi culture but also having some knowledge of Sámi language. The participants emphasised that their non-Sámi colleagues should have the ability to identify Sámi language, know some words in Sámi, and know who to contact if the patient only spoke Sámi. This could be, for instance, if the patient had gone back to their mother tongue because of dementia. One participant said it in the following way:

“There are some older Sámi that go back to their mother tongue if they get dementia, and if nobody has Sámi language skills then it is terrible. The non-Sámi healthcare staff must know what to do then”.

#### 3.1.2. Knowledge and Respect for Diversities within Sámi Cultures

Although all participants stressed that all non-Sámi healthcare personnel should have more general knowledge about Sámi history, culture, and language, they also emphasised the importance of knowing about differences between all patients, whether Sámi or not. This means that healthcare personnel need general knowledge but also knowledge about diversities within communities and specific knowledge about all individual patients. One participant explained the following:

“Sámi culture is a lot, there’s diversity…//… You cannot learn something about Sámi culture and think it’s like that for every Sámi, because there are personalities and diversities within the Sámi community. You have to relate to Sámi people differently, because there’s variety between all patients”.

This statement about “relating to Sami differently…” creates expectations that Sami patients are different from other patients, while at the same time all patients are humans and individuals with their varieties.

The participants also stressed that a lack of general knowledge about Sámi culture, diversities within communities, and specific knowledge about individual patients could lead to challenging situations for Sámi patients. Despite the lack of knowledge among non-Sámi healthcare personnel and obvious general knowledge all healthcare personnel should know about Sámi patients, some participants highlighted that one Sámi is not another Sámi; one must see the uniqueness in every Sámi patient to meet their individual care needs, just like any other patient. One participant said the following:

“It’s important that you have local knowledge, because that’s vital for each individual patient when they need help from us healthcare staff”.

Local knowledge may mean that you know the family history of the patient through your position in the community, which may involve, e.g., Sámi traditions, reindeer husbandry, the person’s life story, or social norms. This was stressed by the participants, who meant that healthcare personnel had to follow every patient’s rhythm, including that of Sámi patients. One participant said the following:

“You must be aware of the lifestyle. Because if you start making dinner for a Sámi patient at one o’clock, maybe he doesn’t want dinner until nine o’clock. So, you must follow the rhythm of the person, we must ‘read people a bit’”.

One aspect was especially stressed by the participants in this study, namely, the need to show respect to Sámi patients. Therefore, although there may be things that non-Sámi healthcare personnel do not know in encounters with Sámi patients, respect for their beliefs, culture, and points of view is essential. Two participants explained this to the interviewer:

“I: What do you think is most important in an encounter with a Sámi patient?

Participant 1: ‘…you must be open and have respect for our differences and that there are different cultures, and we must try to meet the patients’ needs. But we also must see that even if we have the same culture, we’re individuals and Sámi patients differ as well.’

Participant 2: ‘I do agree. Respect. It’s basic, you must have respect for the people you meet, because we can be different. And I think if you have respect for others, and you have that in the back of your mind, then I think you can achieve a lot’”.

### 3.2. More Authentic Encounters When Having the Same Background

#### 3.2.1. Just Knowing Who Is Sámi 

Several participants shared that they could sense that a patient was Sámi, sometimes through small artefacts, even if the patient did not reveal this. In this way, they had the ability to see the patient in a different way because they had knowledge that non-Sámi healthcare personnel do not have. One participant explained this when she saw a bag of coffee hanging that she identified as a Sámi artefact. This led the patient to express his sadness at having suppressed being Sámi all his life.

“I worked at a nursing home in X, and there was a patient on the second floor I noticed without being able to say why. I saw that he had a bag of coffee hanging on his cupboard door, so I started talking about that bag, about what it was. Then he asked, are you a Sámi? Yes, I said. Then he told me about the evacuation from Finnmark and that he had to move. He said nothing about being Sámi, but he said he knew a word in Sámi and it was “eadni” (North Sámi for mother) and then he started to cry. The tears rolled down his cheeks when he said that, and it was something he had repressed and didn’t want to talk about. But he opened up a little bit when I said I was Sámi. And he said to me, you shouldn’t talk about being Sámi. Although he did not want to admit it, there was no doubt that he was Sámi. It was kind of funny, just that little bag of coffee that opened up the conversation”.

Another participant also stated that having the ability to identify who was a Sámi patient as a Sámi healthcare professional could mean that the patient or relatives would receive better healthcare. This could be crucial for understanding and was exemplified by one participant who enabled communication, motivated by expressing ethnic togetherness.

“It’s hard to explain, but you just know when I meet patients that are Sámi…//… There was a family with a severely ill child, and the doctor explained to the family, saying: “Do you understand?” They all nodded. Then I saw that the old grandmother left the room, she couldn’t cope with being there. Then I thought, she hasn’t understood a thing the doctor has said. I followed her, and I sat down with her and then I greeted her in Sámi, and I asked her, “Did you understand?” She said, “No.” And then I spent some time with her. Because then she knew who I was, and we got a different connection. I cannot say why I knew she was Sámi, but I just knew”.

#### 3.2.2. Feelings of Togetherness When Having the Same Background

All participants found that their healthcare encounters with Sámi patients became more authentic because they had the same background. Several explained that the Sámi patients became more comfortable and that the patients said this themselves.

“I very rarely meet a Sámi patient who doesn’t say anything about my being Sámi. It’s often something they point out very quickly, and they say, ‘You’re one of our own’. I can only tell this to you. I wouldn’t say this to anyone else because you understand…//…they have confidence in me. I see it’s sort of something you only experience when you’re from the same culture”.

Another participant gave a further example:

“You have it ‘in your spine’. There’s something about these things you just know. You don’t have to explain, you don’t have to ask, you just know…//… He knows who I am, and where I come from, so that’s where much of the security comes from”.

The expressed “feeling of togetherness” and “you have it in your spine” may be based on cultural inside knowledge such as cultural signs and greeting traditions, both from the language, the way you approach people, the way you talk, and what you talk about. This can be an embodied way to express yourself which gives recognition and the feeling of cultural togetherness.

Several participants also stated that their patients revealed that when they meet non-Sámi healthcare personnel, the personnel do not understand what they are trying to say:

“Many patients say: ‘It’s completely different when you come, you’re our people’. That’s because I get a different connection with them. Others [non-Sámi healthcare personnel] don’t understand and when they don’t understand, they don’t understand…//…They don’t understand spiritual things, feelings, suffering, habits and bad habits, clothes, and everything. It’s so good to be with your own…//… It’s such a natural connection. They are themselves and they feel good and it’s good for them when we who are Sámi come”.

Participants in this study also pointed out that not all Sámi patients were accustomed to meeting Sámi healthcare personnel, so when they did, they appreciated it and expressed their pleasure to the participant, verbally and non-verbally. One said the following:

“They’re very similar to Northern Sámi patients, but they have little experience of meeting Sámi healthcare personnel with expertise in Sámi language and culture, so they’re very relieved when they meet someone with Sámi cultural and language expertise. And I can see they relax more, patients that I haven’t met before and don’t even know who I am…//…they trust me completely differently (when they meet me as a Sámi healthcare professional)”.

## 4. Discussion 

The aim of this study was to explore how Sámi healthcare staff experience encounters with Sámi patients and what expectations they have for non-Sámi healthcare staff. The analysis revealed the themes “various expectations for non-Sámi healthcare staff” and “more authentic encounters when having the same background”. We have chosen to discuss the findings in light of tacit knowledge, two-eyed seeing, and culturally safe care. 

One of the themes, “various expectations for non-Sámi healthcare staff”, demonstrated that the participants expected their non-Sámi colleagues to have more general knowledge about Sámi history, culture, and language but also about diversities within the Sámi communities and between Sámi patients. The expectations for non-Sámi healthcare personnel can be summarised as expectations to provide person-centred and culturally safe care. The results from this study exemplify that it is essential that non-Sámi healthcare staff have knowledge of and acknowledge the historical traumas of the Sami people, as shown in the story about “eadni” (North Sámi for mother) and the neglect of one’s own ethnicity. This is in order to provide person-centred and culturally safe care.

Williams [41] stresses that a culturally safe environment can be created when there is shared respect, meaning, and knowledge in encounters but also learning together with dignity and truly listening. Smye et al. [42] also point out that cultural safety is shaped by, e.g., political, cultural, and social structures. Blix [43] therefore points to the importance of reflecting on our own cultural background as healthcare personnel and the fact that we are influenced by the culture we live in, our education systems, and our previous experiences. In this way, we all have beliefs, values, and norms that have a bearing on how we encounter and care for patients in healthcare settings. Most participants in this study stressed the importance of showing respect to all patients, including Sámi patients, which is expected from healthcare staff in general, as professionals [44]. Having knowledge of Sámi history, culture, and language is therefore essential in order to provide culturally safe care, as highlighted by, e.g., Williams [41]. The concept of culturally safe care is strongly linked to person-centred care; therefore, if healthcare personnel focus on person-centredness in encounters with patients and are respectful and aware of the power position they possess, it will be easier to create a culturally safe care environment for the patients. All patients have a need to be seen as the person they are in relation to their cultural background and in relation to their preferred language in healthcare encounters [29,32]. Using a person-centred approach which includes knowledge of patients’ life story, culture, and family connections can therefore be seen as respecting care recipients’ values and their individual right to self-determination, which will lead to a more respectful encounter between the healthcare provider and recipient, as emphasised by McCormack et al. [45]. This is seen in the findings where the participants expect non-Sámi healthcare staff to have knowledge about Sámi culture and language but also respect for diversities within Sámi cultures. Knowledge of Sámi culture is essential for healthcare personnel, as highlighted in general health policy, legislation, and guidelines in addition to the various national guidelines for healthcare personnel [7,9,27,28]. This means that healthcare facilities and individual healthcare personnel are both responsible for providing culturally safe care, which shows that the expectations of the participants in this study are valid and very legitimate. This is also an argument to prevent cultural load for Sámi staff, who must constantly act as cultural guides between Sámi patients and the rest of the staff [46]. Non-Sámi healthcare personnel must take their share of responsibility to provide culturally safe care to all patients they meet and not leave the responsibility to their Sámi colleagues. A focus on Sámi culture and expectations from Sámi healthcare staff, as demonstrated in this study, have been included in all bachelor’s programmes in healthcare in Norway since 2019 [47]. National guidelines for healthcare personnel also now emphasise the expectations for healthcare staff at the system level [27]. Another issue highlighted by the participants in this study is respect. This involves respect when encountering Sámi patients and respect for what one does not know, as one participant in this study said, “…if you have respect for others, and you have that in the back of your mind, you don’t have to have a similar culture to show respect, and you don’t have to ask either, then I think you can go far”. This quotation also expresses the importance of seeing the uniqueness in every patient, realising that we are all different, and making an effort to treat every patient respectfully. Browne [48] emphasises that respect expressed by non-Indigenous healthcare personnel when encountering Indigenous patients should contain reflected ethical values related to equality, inherent worth, and the uniqueness and dignity of the individual. This does not make the expectations of the participants in this study for their non-Sámi colleagues any less legitimate; non-Sámi healthcare personnel still need to increase their knowledge of Sámi culture and language and the diversities within Sámi culture, in addition to the respect underlined by both Browne [48] and the participants in this study. The participants’ expectations in this study can also be seen as resistance towards the healthcare system and individual non-Sámi healthcare staff members. The participants expect their non-Sámi colleagues to enhance their knowledge of Sámi culture, language, and diversities within the Sámi communities but also to see the uniqueness of the individual patient. They also expect this to be conducted with respect. This can be seen as a form of resistance and an expectation that non-Sámi colleagues have a holistic approach to Sámi patients and their families. Bearskin [49] states that if Indigenous nurses were provided with options to consider and actively explore Indigenous knowledge like the participants in this study, their experiences could support the reawakening of their embodied knowledge [49]. In this study, this could be exemplified by the experiences and expectations the participants have regarding their non-Sámi colleagues. This could therefore make their non-Sámi colleagues take their lack of cultural knowledge seriously and thus enhance their own knowledge and be able to provide more culturally safe care. 

The theme “more authentic encounters when having the same background” shows that the participants in this study felt instant togetherness in encounters with Sámi patients in contrast to non-Sámi patients. The participants often knew who had a Sámi background, even if this was not revealed by the patients themselves. This can be seen as an ability to use the concept of “two-eyed seeing” which was developed by Bartlett et al. [50]. This pertains to the ability to perceive things from two perspectives simultaneously and to utilise these perspectives in one’s professional practice as healthcare personnel. Wright et al. [51] stress that two-eyed seeing has been defined as an equitable approach where shared perspectives and all viewpoints are valued, using the best from each worldview and thus creating a space to learn from each other and appreciate each other’s differences. The holistic approach in “two-eyed seeing” may therefore be seen as trees holding hands (roots) beneath the ground and being together despite our differences [50]. Wright et al. [51] found that the important aspects of “two-eyed seeing” are that it values all perspectives, it is a decolonising approach, and it helps us to learn from each other and understand differences in approaches to health and therefore also in encounters with patients of an Indigenous background. In relation to the results in this study, the participants “just knew” who had a Sámi background but could not always identify why they knew when the interviewer asked about this. In one of the narratives presented, the participant told a story about meeting an old patient in a nursing home. The encounter enabled the participant to sense that the patient was Sámi because a bag of coffee, a Sámi artefact, was hanging there, and she instantly felt a connection to the patient, who had for many years suppressed his Sámi identity. Having the ability to use her Sámi perspectives when she encountered the Sámi patient highlights the importance of the concept of “two-eyed seeing”. The participant would also be more likely to provide culturally safe care, having identified the patient’s background, even though he did not himself reveal this directly. This made it easier to provide culturally appropriate and safe care, especially in this case, considering the trauma after the Norwegianisation process that probably led to the patient’s suppression of his Sámi identity, which applies to many Sámi. Willams et al. [29] state that culturally safe care can be experienced as spiritually, socially, emotionally, and physically safe. The importance of “two-eyed seeing” is also revealed in another story where the participant identified a Sámi grandmother who left the hospital room when the doctor explained the condition of her severely ill grandchild. Being a Sámi herself, the participant instantly realised that the grandmother was a Sámi and had probably not understood the doctor’s information and therefore followed her when she left the room. By identifying the grandmother as Sámi, she had the ability to explain the information given and was thus more likely to provide culturally safe care. The instant togetherness the participants in this study felt with Sámi patients can be interpreted as being accepted and recognised as being among “our people”, resulting in culturally safe encounters for the patients, who know that somebody understands their culture and where they “come from”. This can also be seen in relation to having tacit knowledge, which can be defined as knowledge people carry in their body and mind, which is therefore difficult to access and share [52]. When the participants in this study encountered Sámi patients, they sensed who was Sámi and therefore knew how to relate to them. As one participant said, “You have it in your spine. There’s something about these things you just know”, and this may be something all healthcare personnel must acknowledge when striving to provide culturally safe care to all patients. Having the same cultural background is an advantage, but in any case, all healthcare personnel must make efforts to ensure that all their patients receive culturally safe care.

## 5. Methodological Considerations

This study aimed to provide insight into a specific phenomenon through several focus groups and individual interviews, and this is one of the strengths of qualitative research. Although the results cannot show the experiences of all healthcare personnel with an Indigenous background, they may have transfer value to other contexts of healthcare personnel of Indigenous background and their Indigenous patients. In this study, our original intention was not to use both individual and focus group interviews. Lambert and Loiselle [53] point out that combining individual and focus group interviews could be a way to enhance data richness. We have seen in our study that using both approaches has enriched the data through longer and more in-depth interviews with the participants. We have therefore explained this in detail in the methodology section to achieve transparency. Both authors also participated in the analysis and had broad discussions to achieve a consensus on the results presented in this study. Another limitation could be that interviews were conducted in Norwegian, but this was conducted because the participants spoke different Sámi languages, and some of the participants did not speak Sámi, and neither did the interviewer. This shows that Norwegian is the predominant language in healthcare services. 

## 6. Conclusion and Implications for Practice

The aim of this study was to explore Sámi healthcare staff experiences in encounters with Sámi patients and their expectations for non-Sámi healthcare staff. This study shows that the participants expected non-Sámi healthcare staff to have knowledge about Sámi history, culture, and language, in addition to knowledge and respect for diversities within Sámi cultures. The findings in this study can be seen as a form of resistance by the participants but which can be addressed by non-Sámi healthcare staff and the overall healthcare system. This implies that all healthcare personnel must have knowledge of Sámi history, culture, and language and knowledge and respect within Sámi cultures to see the uniqueness in every patient’s life story; they must therefore respect each patient they encounter by providing culturally safe care. Non-Sámi healthcare personnel and the overall healthcare system should thus meet the expectations expressed by the participants in this study and enhance their knowledge of Sámi history, culture, and language in order to ensure culturally safe care. This can succeed if departments of universities follow the new guidelines focusing on Sámi knowledge in their education and leaders in primary and secondary healthcare provide courses in Sámi culture to ensure culturally safe care for all patients. Further, tacit knowledge and “two-eyed seeing”, as demonstrated by the participants in this study, are not necessarily transferable to non-Sámi healthcare personnel. Despite this, all healthcare personnel, through experience, recognition, and self-reflection on encounters with Sámi patients and Sámi healthcare personnel, can develop their knowledge and skills within the concept of “two-eyed seeing”. 

## Figures and Tables

**Table 1:** Results of analysis.

Various expectations for non-Sámi healthcare staff	Knowledge about Sámi culture and language
	Knowledge and respect for diversities within Sámi cultures
More authentic encounters when having the same background	Just knowing who is Sámi
	Feelings of togetherness when having the same background

## Data Availability

The data are in Norwegian and not available for international readers.

## Public Involvement Statement

There was no public involvement in any aspect of this research.

## Guidelines and Standards Statement

The consolidated criteria for reporting qualitative research (COREQ) were used in relation to this study.
